# Molecular characterization of free fatty acid receptors FFAR2 and FFAR3 in the domestic cat

**DOI:** 10.1002/vms3.356

**Published:** 2020-09-15

**Authors:** Ichiro Yamamoto, Koh Kawasumi, Kozo Ohkusu‐Tsukada, Toshiro Arai

**Affiliations:** ^1^ Department of Basic Veterinary Medicine School of Veterinary Medicine Faculty of Veterinary Science Nippon Veterinary and Life Science University Musashino‐shi Tokyo Japan; ^2^ Department of Veterinary Pathology School of Veterinary Medicine Faculty of Veterinary Science Nippon Veterinary and Life‐Science University Musashino‐shi Tokyo Japan

**Keywords:** cat, FFAR2/GPR43, FFAR3/GPR41, short chain fatty acid

## Abstract

G protein‐coupled receptors 41 and 43 were identified and characterized as free fatty acid receptors (FFAR) 3 and 2, respectively. FFAR2 and FFAR3 mediate short‐chain fatty acids (SCFAs) as signalling molecules. The present study aimed to give molecular characterization of FFAR2 and FFAR3 in the domestic cat. High homology with that in other mammals was revealed by cDNA cloning of cat FFAR2 FFAR3. We analyzed the tissue distribution of cat FFAR2 and FFAR3 mRNA using quantitative polymerase chain reaction. The inhibition of intracellular cAMP concentrations was observed in cells transfected with cat FFAR2 or FFAR3 and treated with SCFAs. The activation of nuclear factor of activated T cells‐luciferase reporter was only observed in cat FFAR2 transfected cells but not in FFAR3. Split luciferase assay (NanoLuc Binary Technology; NanoBiT) for FFAR2 or FFAR3 and Arrestin‐3/β‐arrestin‐2 revealed acetate‐/propionate‐induced recruitment to cat FFAR2 or FFAR3 in CHO‐K1 cells. Our results indicate that FFAR2 and FFAR3 are functional receptor proteins that are expressed in cat tissues and show differential distribution patterns.

## Graphical abstract

Short‐chain fatty acids (SCFAs) are essential nutrients that act as signalling molecules in various cellular processes and are mediated by free fatty acid receptors (FFARs). In the cat, SCFAs are produced via microbial fermentation in the colon. The present study aimed to give molecular characterization of FFAR2 and FFAR3 in the domestic cat.

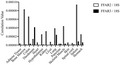

## 1 INTRODUCTION

More than 70 different fatty acids are classified by their carbon chain length (Yonezawa et al., 2013), including short (C2–C6), medium (C7–C12) and long‐chain fatty acids (>C12) (Hara et al., 2014). Short‐chain fatty acids (SCFAs) are produced via fermentation of fibres and digestion of dietary carbohydrates in the gut. In ruminant animals, SCFAs from microbial fermentation are an important energy source, and nonruminant animals consume SCFAs as an energy source. Although cats are carnivorous animals but total SCFAs production in vitro by faecal microflora was greatest when compared to dogs, horses, humans and pigs (Sunvold, Hussein, Fahey, Merchen, & Reinhart, 1995). In addition, adaptation of dietary fibres significantly increased the production of SCFAs by diluted cat faeces in vitro (Barry et al., 2011). It has been reported that adult cats fed commercial laboratory dry diet also produced SCFAs in the gastrointestinal tracts (Brosey, Hill, & Scott, 2000).

SCFAs are essential nutrients that act as signalling molecules in various cellular processes (Kimura et al., 2011). They are mediated by free fatty acid receptors (FFARs) including GPR40/FFAR1, GPR41/FFAR3, GPR43/FFAR2, GPR84 and GPR120/FFAR4 (Yonezawa et al., 2013). FFARs1–4 and GPR84 have been reported to be orphan G protein‐coupled receptors in genomic DNA or cDNA sequences (Fredriksson, Höglund, Gloriam, Lagerström, & Schiöth, 2003; Sawzdargo et al., 1997; Wittenberger, Schaller, & Hellebrand, 2001). FFAR1 was characterized as a middle‐ and long‐chain fatty acid receptor, and free fatty acids (FFAs) amplified glucose‐stimulated insulin secretion from pancreatic β‐cells via activation of FFAR1 (Itoh et al., 2003). FFAR4 was also characterized as a long‐chain fatty acid receptor that mediates gut glucagon like peptide‐1 secretion in colonic intraepithelial neuroendocrine cells (Hirasawa et al., 2005). Human GPR42 is considered a pseudogene, and it differs from GPR41/FFAR3 by only six amino acid residues (Brown et al., 2003). GPR84 was shown to bind to medium‐chain fatty acids and mediate interleukin‐4 gene expression in activated T cells (Venkataraman & Kuo, 2005; Wang, Wu, Simonavicius, Tian, & Ling, 2006). FFAR2 was identified as an SCFA receptor that controls insulin sensitivity and fat accumulation (Brown et al., 2003; Kimura et al., 2013; Le Poul et al., 2003), whereas FFAR3 was identified as an SCFA receptor that regulates the host energy balance via gut microbiota (Brown et al., 2003; Samuel et al., 2008).

Using quantitative polymerase chain reaction (qPCR), cat FFAR1 and FFAR4 were cloned and their cDNAs analysed for mRNA expression patterns (Habara et al., 2015). FFAR1 mRNA was found to be expressed in the duodenum, liver and pancreas, and high levels of FFAR4 mRNA were expressed in adipose tissue. In the cat, FFAR1 and FFAR4 mRNA are differentially controlled by mRNA expression mechanisms. The present study aimed to give molecular characterization to FFAR2 and FFAR3 in the domestic cat.

## 2 MATERIALS AND METHODS

### 2.1 cDNA cloning of cat FFAR2 and FFAR3

Cat tissue total RNA was purchased from Zyagen. This cat tissue was obtained from veterinary clinic and hospitals or certified animal tissue banks in USA. Tissues are freshly harvested under strict regulation by veterinarian during surgical operation or from animals donated for scientific research with the owner consent. A cDNA library was synthesized from duodenum total RNA using the SMARTer RACE cDNA amplification kit (Clontech). The 5’‐ends of FFAR2 and FFAR3 were amplified using the antisense primers FFAR2‐A1 (5’‐GGCAGATACCAGCGGAAGTTATAGG‐3’) and FFAR3‐A1 (5’‐GGTGGCTGTAGCAGTAGATGGTGAT‐3’). The 3’‐ends of FFAR2 and FFAR3 were amplified using the sense primers FFAR2‐S1 (5’‐CCCCCTGCTCTTCTACTTCTCTTCA‐3’) and FFAR3‐S1 (5’‐GAGATGGCTGTGGTCCTTTT‐3’). Primers were designed from predicted cat FFAR2 (XM_003997914) and FFAR3 (NC_018737) sequences. PCR products were purified and cloned into T‐Vector pMD19 (Takara), and sequences were determined using Applied Biosystems 3,130 × l. The complete FFAR2 sequence was combined and determined with 5’, 3’‐RACE, and expression vector of FFAR2.

### 2.2 Quantitative realtime PCR (Q‐PCR) for cat FFAR2 and FFAR3

Total RNA was reverse‐transcribed using the PrimeScript reverse transcription (RT) reagent kit with a gDNA Eraser kit (Takara). Genomic DNA was removed from the total RNA sample before cDNA synthesis. The cDNA was used as template DNA for RT‐PCR, and total reaction volumes of 20 μl were composed of 1× TB Green Premix EX Taq II (Takara) containing 0.625 U Ex Taq HS, 200 μM dNTP, and 0.2 μM of each primer (FFAR2–S2: 5′‐GGACCTCCTGCTGCTGCT‐3′, FFAR2–A2: 5′‐AGCATCTACTGCAGCACGTG‐3′; FFAR3–S2: 5′‐GAGATGGCTGTGGTCCTTTT‐3′, FFAR3–A2: 5′‐GCTCAACTTCCTCGTCTGCT‐3′). The qPCR was performed as follows: predenaturation at 94°C for 2 min, 40 cycles of denaturation at 94°C for 10 s and annealing and extension at 60°C for 35 s. After the qPCR reaction, melting curve analysis was performed to check the specificity of the qPCR product. Quantitative analysis was conducted using a series of plasmid DNA dilutions. Expression levels of 18S ribosomal RNA were used as internal controls and measured by qPCR using primers for 18S‐S (5′‐GTAACCCGTTGAACCCCATT‐3′) and 18S‐A (5′‐CCATCCAATCGGTAGTAGCG‐3′).

### 2.3 Construction of the expression vector for cat FFAR2 and FFAR3

A cDNA library was appropriated from the 5′ RACE cDNA library of cat duodenum. The open reading frame (ORF) region of cat FFAR2 and FFAR3 was amplified by PCR using FFAR2‐S3 (5′‐cagtgtggtggaattATGACAAACTGGCGCAGCTC‐3′) and FFAR2‐A3 (5′‐tagactcgagcggccCTAGTCTGTAGTGAAGTCGG‐3′), FFAR3‐S3 (5′‐cagtgtggtggaattATGGACACCAGCCCGGACCG‐3′) and FFAR3‐A3 (5′‐tagactcgagcggccCTACGTTGGACAGCTCCCCC‐3′). Lowercase letters indicate the vector overlap regions for InFusion HD cloning into the *EcoRI–NotI* site of pcDNA3.1/V5–HisB (Invitrogen) using the InFusion HD cloning kit (Clontech Takara) and sequenced.

### 2.4 GloSensor cAMP assay for cat FFAR2 and FFAR3

We monitored the changes to intracellular cAMP levels using a functional assay for cat FFAR2 or FFAR3 in CHO‐K1 cells. Because FFAR2 and FFAR3 are classified in G_αi_‐coupled G protein–coupled receptor (GPCRs), we used forskolin as an adenylate cyclase activator. Cat FFAR2 or FFAR3 expression vectors were transfected into CHO‐K1 cells with pGloSensor‐22F cAMP plasmid (Promega). CHO‐K1 cells were seeded at a density of 5 × 10^5^ cells/well on six‐well plates for 24 hr. Cells were subsequently transfected with 625 ng of cat FFAR2 or FFAR3 vectors and 625 ng of pGloSensor‐22F cAMP plasmid using 6.25 μl ScreenFect A (Fujifilm Wako Pure Chemical). At 24 hr after transfection, cells were reseeded at a density of 10,000 cells per well on a 384‐well plate for 18 hr. The transfection efficiency of FFAR2 and FFAR3 expression vectors revealed 38% and 34% in CHO‐K1 cells, respectively. Media were changed to Ham's F12 medium containing 0% fetal bovine serum (FBS) and 6% GloSensor cAMP reagent (Promega) for 2 hr at room temperature. Reseeded CHO‐K1 cells were pretreated with varying concentrations of FFAs for 5 min before treatment with 10 μM forskolin. Luminescence was measured using a GloMax Explorer Multimode Microplate Reader (Promega) 30 min after forskolin addition, and this value (*n* = 6) was represented as a 100% value to determine each of the pEC_50_ values.

### 2.5 NFAT‐luciferase reporter assay for cat FFAR2 and FFAR3

We performed nuclear factor of activated T cells (NFAT) luciferase reporter assay was used to measure Ca^2+^ signalling via stimulation of cat FFAR2 or FFAR3. CHO‐K1 cells were seeded at a density of 5 × 10^5^ cells/well on six‐well plates for 24 hr. Cells were subsequently transfected with 625 ng of cat FFAR2 or FFAR3 vectors with 625 ng of pGL4.30 (luc2P/NFAT/Hygro) and 10 ng of pNL1.1 PGK (Nluc/PGK) plasmid using 6.25 μl ScreenFect A (Fujifilm Wako Pure Chemical). At 24 hr after transfection, cells were reseeded at a density of 10,000 cells per well on a 384‐well plate for 18 hr. Media were changed to Ham's F12 medium containing 0% FBS and varying concentrations of acetate for 5 hr. Luminescence was measured using a Nano‐Glo Dual luciferase reporter assay system (Promega) and this value (*n* = 4) was represented as a ratio of NFAT/ NLuc value to determine each of the pEC_50_ values.

### 2.6 NanoBiT luciferase assay to measure protein‐protein interaction

NanoBiT split luciferase assays were used to observe FFAR2 or FFAR3 and Arrestin interactions according to the manufacturer's protocol. Expression vectors were prepared by PCR cloning of the ORF regions of cat FFAR2, FFAR3, Arrestin‐1/SAG (GenBank accession number XM_003991270), Arrestin‐2/ARRB1 (GenBank accession number XM_006936921), Arrestin‐3/ARRB2 (GenBank accession number XM_006939731), and Arrestin‐4/ARR3 (GenBank accession number XM_019823924). Other than cat FFAR2 and FFAR3, the arrestin primers used were as follows: Arrestin‐1 sense (5′‐ATGGCGGCCAGCGGGAAGAC‐3′), Arrestin‐1 antisense (5′‐TCCGCGGCCTCCC‐3′), Arrestin‐2 sense (5′‐ATGGCTTCCCCGTTCCTGA‐3′), Arrestin‐2 antisense (5′‐TCTGTCGTTGAGCTGT‐3′), Arrestin‐3 sense (5′‐ATGGGGGAGAAACCGGGCA‐3′), Arrestin‐3 antisense (5′‐GCAGAACTGGTCCTCGTAA‐3′), Arrestin‐4 sense (5′‐ATGGCCAACATGTCAAGGGT‐3′) and Arrestin‐4 antisense (5′‐GCTTCCCTCATCCCCCT‐3′). The gene ORF regions were cloned into pFN33K LgBiT TK–Neo Flexi, pFC34K LgBiT TK–Neo Flexi, pFN35K SmBiT TK–Neo Flexi, and pFC36K SmBiT TK–Neo Flexi vectors according to the manufacturer's protocol. Before starting the FFAR2 or FFAR3–Arrestin interaction assay, all possible combinations of fusion protein pairs were tested (N‐ or C‐terminal fusion of split luciferase), and the fusion protein pair that provided the brightest relative signal was selected for assay. To confirm specific FFAR2 or FFAR3–Arrestin interactions, HaloTag–LgBiT (NanoBiT‐negative control vector) was compared with luminescence values (>10 times). CHO‐K1 cells were seeded at a density of 4,000 cells/well in 384‐well plates and cultured in Ham's F12 media containing 10% FBS for 24 hr. Cells were transfected with 7.5 ng of LgBiT‐fused Arrestins vectors and 7.5 ng SmBiT‐fused FFAR2 or FFAR3 vectors with 0.075 μl of ScreenFect A (Fujifilm Wako Pure Chemical). Media were changed 24 hr after transfection to Ham's F12 containing 0% FBS for 2 hr to serum‐starve the cells before processing for luminescence. Media were changed to 30 μl of Ham's F12 containing 0.375 μl of Nano‐Glo live cell substrate and 7.125 μl of Nano‐Glo LCS dilution buffer (Promega). Luminescence was measured using a GloMax Explorer Multimode Microplate Reader (Promega). The area under the curve was calculated using GraphPad PRISM 8 (GraphPad PRISM Software) to evaluate the effect of FFAs.

## 3 RESULTS

### 3.1 cDNA cloning of cat FFAR2/GPR43 and FFAR3/GPR41

**FIGURE 1:**
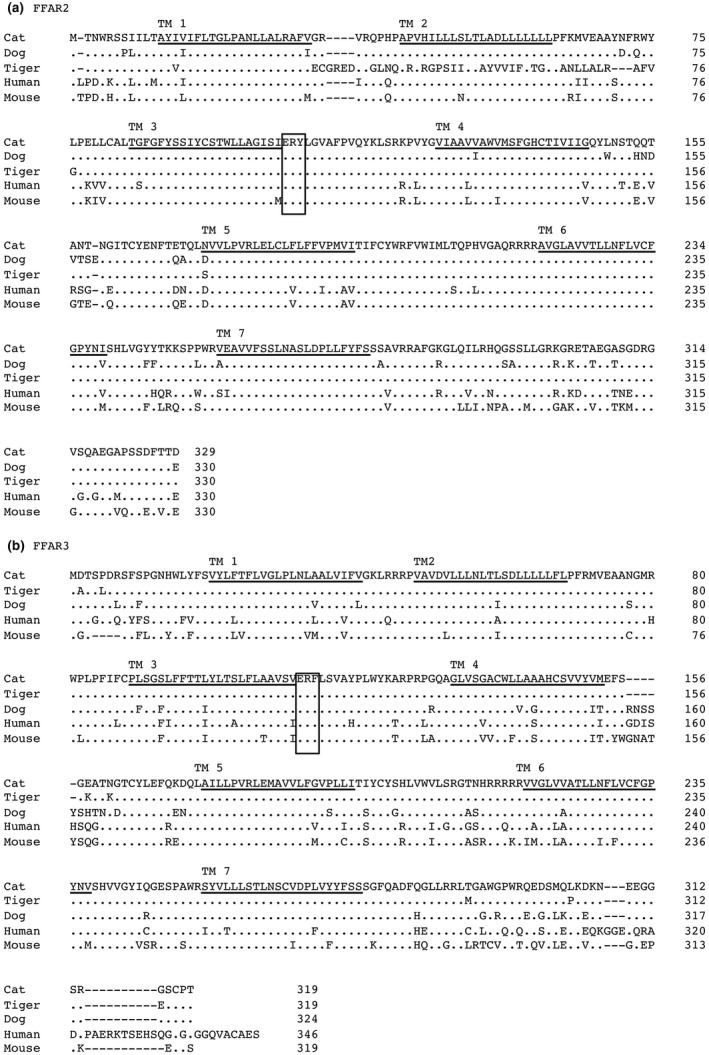
Alignment of the amino acid sequences of cat FFAR2 (a) and FFAR3 (b) with that of other mammalian amino acid sequences. The amino acid sequences of cat FFAR2 (LC500832) were aligned with that of dog (XM_850388), amur tiger (Tiger: XM_015544160), human (NM_005306), and mouse (NM_001168512) FFAR2. Cat FFAR3 (LC500831) was aligned with that of amur tiger (Tiger: XM_007099153), dog (NC_006583), human (NP_005295) and mouse (NP_001028488) FFAR3. The transmembrane regions are underlined. Identical amino acid residues are represented as dots. ERY or ERF motifs are indicated by a double underline

Complete sequence data for cat FFAR2 and FFAR3 cDNAs were submitted to DDBJ/EMBL/GenBank databases (registration accession numbers LC500832 and LC500831, respectively). Cat FFAR2 consisted of 110 bp of the 5′‐untranslated region (UTR), 987 bp of the coding region and 761 bp of the 3′‐UTR. Cat FFAR3 consisted of 29 bp of the 5′‐UTR, 957 bp of the coding region and 547 bp of the 3′‐UTR. A computer‐assisted search for cat FFAR2/GPR43 and FFAR3/GPR41 cDNA sequences in the cat genome (www.ncbi.nlmnih.gov/BLAST) showed that both cat FFAR2 and FFAR3 consisted of two exons. A potential polyadenylation signal was present close to the 3′‐ends of both cDNAs. The amino acid sequences deduced from cloned cat FFAR2 displayed high overall sequence identity to that of dog (90%), amur tiger (87%), human (82%) and mouse (82%). Cat FFAR3 also showed high overall sequence identity to that of amur tiger (97%), dog (87%), human (78%) and mouse (74%) (Figure 1a,b). The ERY and ERF motifs in FFAR2 and FFAR3, respectively, are highly conserved among class A GPCRs and play an important role in ligand binding (Rhee, Nevo, Levy, & Vogel, 2000).

### 3.2 Tissue distribution of cat FFAR2 and FFAR3 mRNAs

**FIGURE 2:**
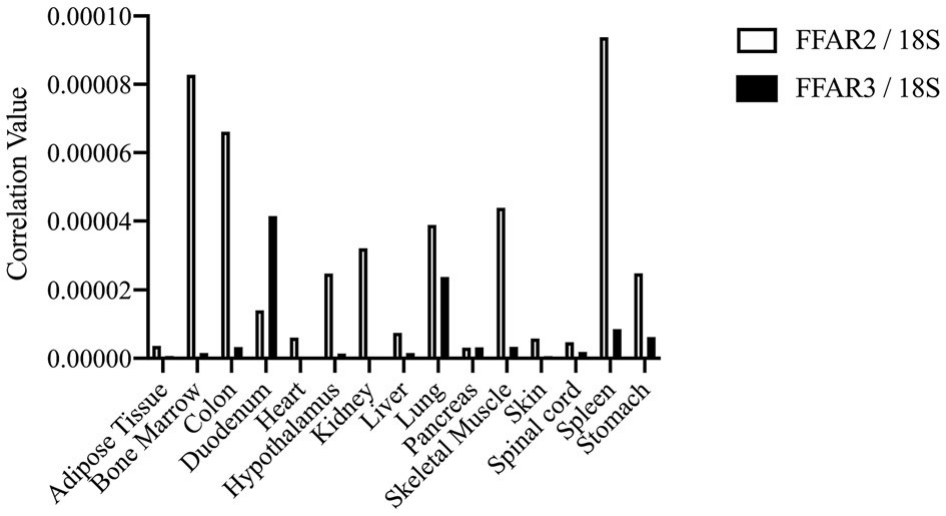
Tissue distribution of FFAR2 and FFAR3 mRNA in the adult cats. FFAR2 and FFAR3 mRNA expression patterns in adult cat tissues were determined by qPCR analysis. Expression levels of FFAR2 and FFAR3 are represented as values corrected to 18S ribosomal RNA. White bars represent FFAR2; black bars represent FFAR3

To determine gene‐ and tissue‐specific mRNA expression profiles of cat FFAR2 and FFAR3, we conducted qPCR analysis using cDNAs derived from adult cat tissues. High levels of FFAR2 mRNA expression were observed in bone marrow, colon and spleen, whereas high levels of FFAR3 mRNA expression were observed in the duodenum and lung (Figure 2).

### 3.3 Measurement of cAMP content and NFAT‐luciferase activity in CHO‐K1 cells expressing cat FFAR2 and FFAR3

**FIGURE 3:**
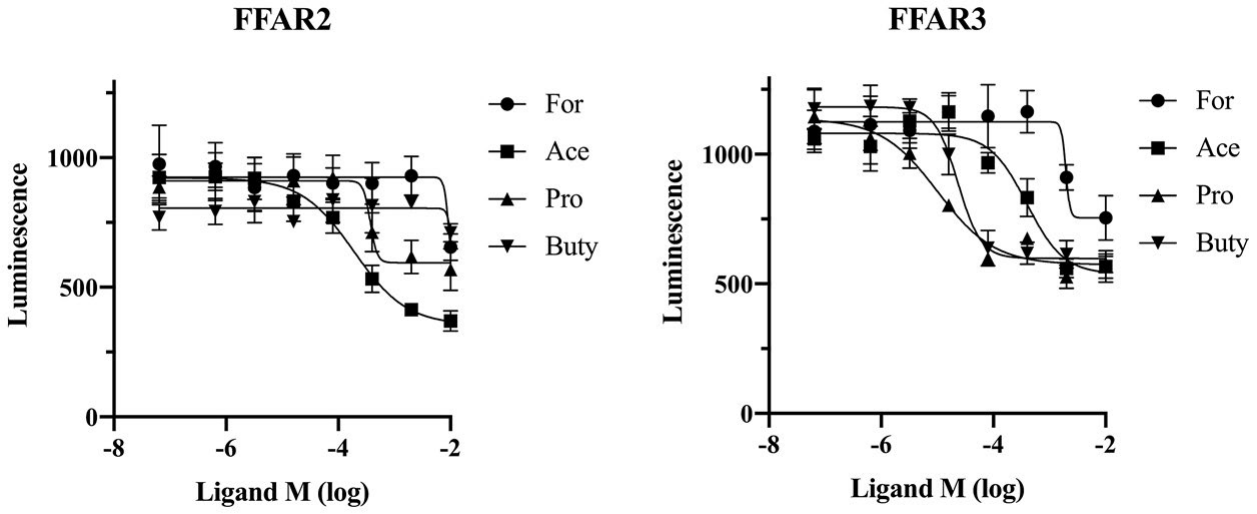
Functional assay for cat FFAR2 and FFAR3. The inhibition of 10 μM forskolin‐induced cAMP increase by acetate (circles), formate (squares), propionate (triangles) and butyrate (reverse triangles) was determined in CHO‐K1 cells transiently transfected with cat FFAR2 or FFAR3 and pGloSensor‐22F cAMP plasmid. Values are expressed as mean ± *SEM* (*n* = 6)

**FIGURE 4:**
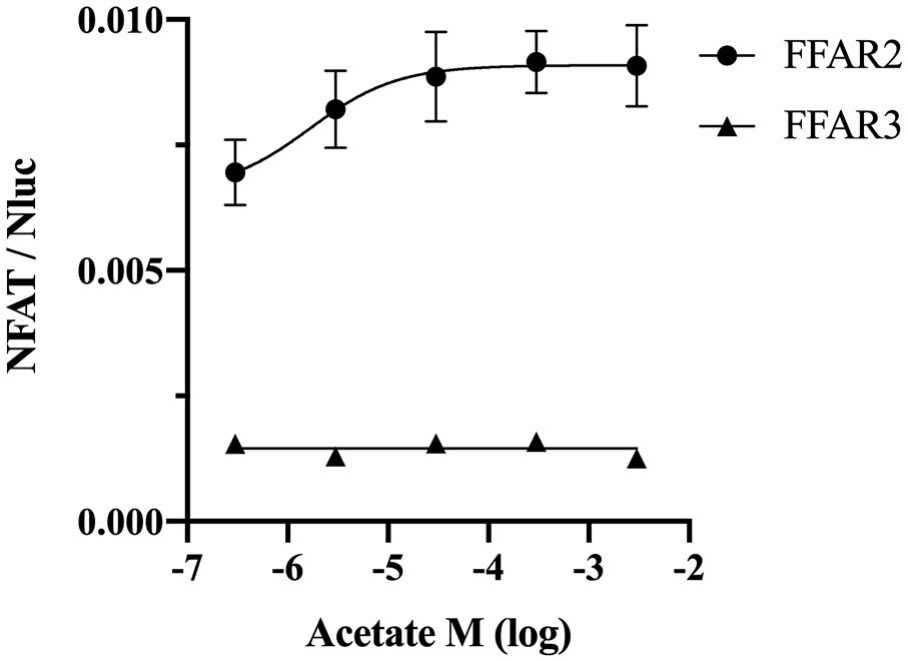
NFAT‐luciferase reporter assay for cat FFAR2 and FFAR3 expressed in CHO‐K1 cells by acetate treatment. The stimulation of NFAT‐Luciferase activity was determined in CHO‐K1 cells transiently transfected with cat FFAR2 or FFAR3 and pGL4.30 (luc2P/NFAT/Hygro) plasmid. Values are expressed as mean ± *SEM* (*n* = 4)

FFAR2 have been previously classified in other animals as G_αi/o_ and G_αq_ receptor, while FFAR3 mainly to G_i/o_ (Sawzdargo et al., 1997; Xiong et al., 2004). However, it is unclear whether cat FFAR2 and FFAR3 can regulate the intracellular signalling. We measured cAMP concentrations in chinese hamster ovary cells (CHO‐K1) transiently expressing cat FFAR2 or FFAR3 using the GloSensor cAMP assay system. We found that increased cAMP levels were directly proportional to increased luminescence resulting from the enzymatic breakdown of the substrate, D‐luciferin (Binkowski et al., 2011). Cells were preincubated with varying concentrations of SCFA for 5 min, followed by 10 μM forskolin stimulation for 30 min. Acetate and propionate inhibited forskolin stimulation in a dose‐dependent manner, and high‐dose formate and butyrate inhibited cAMP accumulation for cat FFAR2 (Figure 3). As compared with FFAR2, FFAR3 was activated by all four SCFA with large pEC_50_ values (Table 1). Also, NFAT‐luciferase activity was measured by acetate treatment because mammalian FFAR2 is known as promiscuous receptor that couples to both G_αi/o_ and G_αq_. Acetate stimulated NFAT‐luciferase reporter activity that transiently transfected cat FFAR2 with pEC50 value (6.141 ± 0.5932) but not FFAR3 (Figure 4).

**TABLE 1:** Values of pEC50 for SCFA inhibition to cAMP accumulation in CHO‐K1

	Formate (C1)	Acetate (C2)	Propionate (C3)	Butyrate (C4)
FFAR2	<2	3.721 ± 0.237	3.430 ± 0.378	<2
FFAR3	2.711 ± 0.128	3.404 ± 0.163	4.994 ± 0.206	4.633 ± 0.115

Abbreviations: FFAR, free fatty acid receptor; SCFA, short‐chain fatty acid.

### 3.4 Desensitization of cat FFAR2 and FFAR3 by SCFAs

**FIGURE 5:**
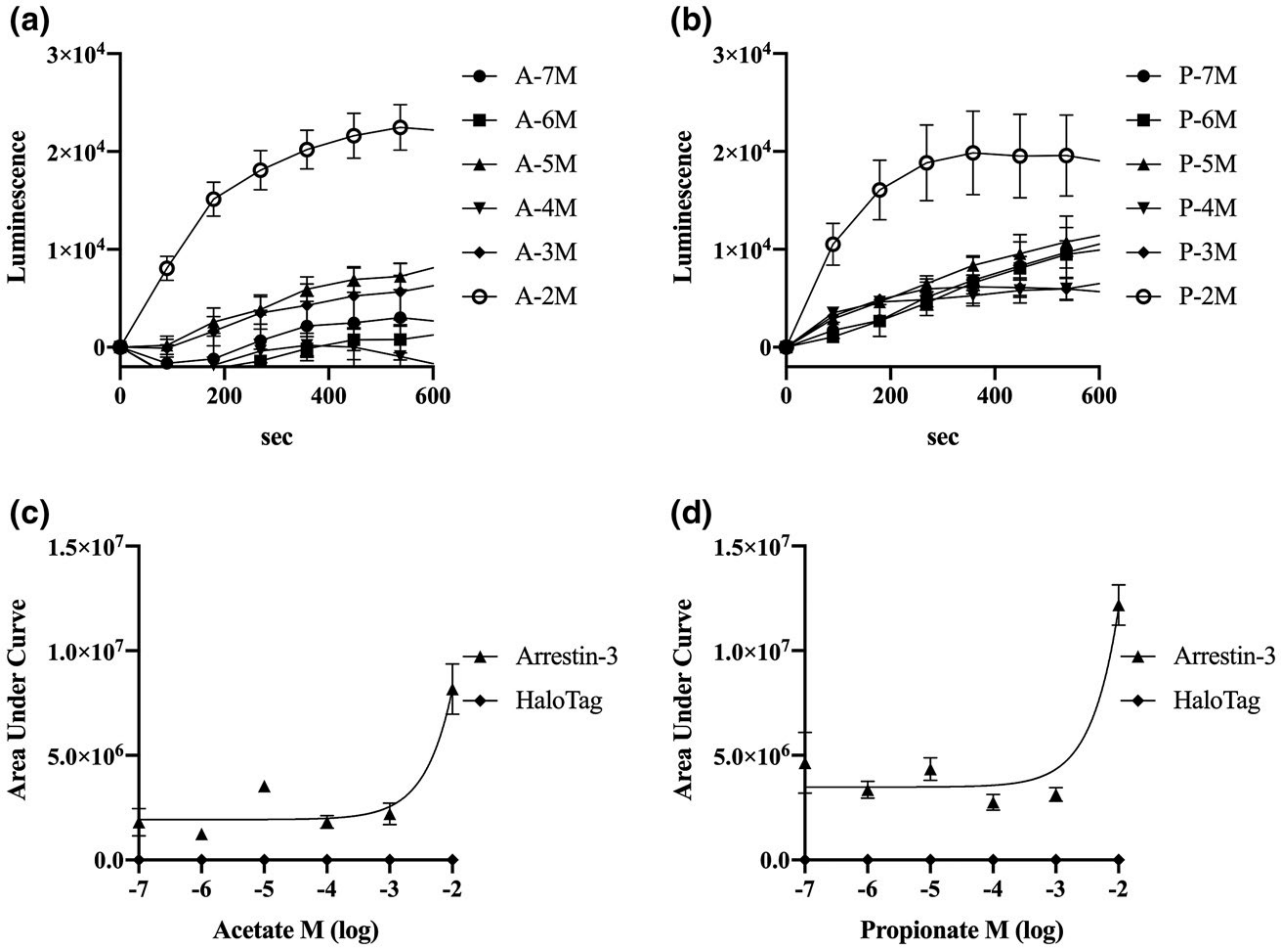
Transient interaction of Arrestin‐3 and cat FFAR2 or FFAR3 expressed in live cells. The interaction of Arrestin‐3 and FFAR2 or FFAR3 after treatment for 0 s with acetate (a: FFAR2, 1 × 10^−2 ~ −7^ M) or propionate (b: FFAR3, 1 × 10^−2 ~ −7^ M). Luminescence was detected as signals and recorded for 10 min, represented as the area under the curve for FFAR2 (c) and FFAR3 (d). Values are expressed as mean ± *SEM* (*n* = 4)

Ligands bind to GPCRs, which then mediate cell‐signalling molecules such as cAMP, inositol phosphates and Ca^2+^ (Srivastava, Gupta, Gupta, & Shukla, 2015). Because continuous cellular signalling by ligand is disadvantageous to cell physiology, GPCR desensitization is an essential process. To understand cat FFAR2 and FFAR3 desensitization, we analyzed the interactions between FFAR2 or FFAR3 and Arrestins 1–4 using a NanoBiT luciferase assay (data not shown). Only Arrestin‐3 and cat FFAR2 or FFAR3 demonstrated a specific interaction, because the luminescence level was more than 10‐fold higher than that of FFAR2 or FFAR3 with HaloTag negative control. Arrestin‐3 was classically identified as ß‐Arerstin‐2 previously. An increase in the FFAR2 or FFAR3 interaction with Arrestin‐3/ß‐Arerstin‐2 was observed during high concentrations of SCFA binding (Figure 5).

## 4 DISCUSSION

In this study, we aimed to molecularly characterize cat FFAR2 and FFAR3. Cloning of cat FFAR2 and FFAR3 cDNA revealed high similarity to that in other animals; however, the region from 33E to 77G of amur tiger FFAR2 showed very low similarity to that of other FFAR2 TM2 regions. The region from 33E to 50P demonstrated very low similarity, whereas the region from 51S to 77G matched that of 7S to 32V of FFAR2. This discrepancy should be elucidated by reanalysis or resequencing of FFAR2 using amur tiger genomic DNA or cDNA. The ERY/DRY motif located in the third transmembrane domain of FFAR2 is one of the most characteristic motifs of the rhodopsin GPCR, and it must be maintained in an inactive state (Fredriksson et al., 2003). An ERF motif, rather than an ERY/DRY motif, was conserved in all mammalian FFAR3, which appeared to have the same function, as the tyrosine residue mutations showed no or only a slight effect on receptor function (Rovati, Capra, & Neubig, 2007).

Different mRNA expression patterns were demonstrated in cat FFAR2 and FFAR3 in the examined tissues. We observed high levels of cat FFAR2 mRNA expression in immune tissues, such as bone marrow and spleen. Before the deorphanizing of FFAR2/GPR43, FFAR2 was considered as an immune signal transducer because of the expression in human leukocytes (Senga et al., 2003). FFAR2 was expressed in mouse bone marrow neutrophils as well, and it mediated neutrophil chemotaxis by SCFA (Vinolo et al., 2011). These reports may account for FFAR2’s characteristic mRNA expression in cat bone marrow and spleen. In digestive organ and other tissues, FFAR2 and FFAR3 expression mechanisms were well characterized in some species. High levels of FFAR2 mRNA expression were observed in mouse stomach, colon, spleen, and adipose tissue, as well as human peripheral blood leukocytes and spleen (Hong et al., 2005; Nilsson, Kotarsky, Owman, & Olde, 2003). In chicken, FFAR2 mRNA was expressed in the testes, spleen, peripheral blood mononuclear cells, adipose tissue, duodenum, lung and liver (Meslin et al., 2015). High levels of FFAR3 mRNA were expressed in the duodenum and lung in cats; kidney, colon and spleen in mice (Hong et al., 2005); and peripheral blood mononuclear cells, polymorphonuclear cells, lung, and adipose tissue in humans (Le Poul et al., 2003). FFAR2 and FFAR3 were also expressed in the rumen epithelium of young and adult cattle (Wang, Akers, & Jiang, 2012; Yang, Zhan, Ning, Jiang, & Zhao, 2020; Zhang et al., 2018). Sensing of SCFAs is an essential function for FFAR2 and FFAR3, and deficiency each of receptors result in chronic inflammation and obesity in mice (Ang et al., 2016; Kim, Kang, Park, Yanagisawa, & Kim, 2013; Yonezawa et al., 2013). SCFAs produced from digestive organs are an indispensable energy source, and these must be sensed by FFAR2 or FFAR3 in ruminant and nonruminant animals.

The functional assays of cat FFAR2 and FFAR3 showed that SCFAs inhibited cAMP accumulation after forskolin treatment of FFAR2‐ or FFAR3‐expressing cells. These results indicate that cat FFAR2 and FFAR3 proteins encoded by the respective cDNAs function as a G_αi_ receptor. Interestingly, the effects of SCFA were significantly different between cat FFAR2 and FFAR3. Acetate and propionate were the most effective ligands for cat FFAR2 and FFAR3, respectively. Brosey et al., (2000) reported each of 19, 52, 57 mmol/L of acetate in luminal contents of adult cat duodenum, proximal and distal colon, respectively. Propionate was detected range from 2 to 27 mmol/L, and butyrate was detected range from 2 to 15 mmol/L in the same luminal content. However, butyrate strongly inhibited cAMP accumulation in FFAR3 as compared with FFAR2 (Figure 3). Butyrate was reported to be a natural, strong ligand for both FFAR2 and FFAR3 in humans (Le Poul et al., 2003) and mouse (Nilsson et al., 2003), but butyrate may have a species‐specific effect on FFAR2 because the inhibition of cAMP accumulation was not followed in a dose‐dependent manner in cattle (Wang, Gu, Heid, Akers, & Jiang, 2009). FFAR2 inhibits cAMP accumulation by SCFAs, while FFAR2 stimulates phospholipase C activity as a G_αq_ receptor (Brown et al., 2003; Le Poul et al., 2003; Nilsson et al., 2003). Our results revealed the possibility that cat FFAR2 can act as G_αi/o_ and G_αq_ receptor for SCFA. It may be suggested that FFAR2 and FFAR3 are classified as same SCFA biosensors, but it is clear that cats also differ in their ligands specificity and signalling pathway.

GPCR desensitization mechanisms are tightly regulated by GPCR kinase and arrestin. Following phosphorylation of GPCR by GPCR kinase, arrestin binds and mediates GPCR internalization to terminate G_αi_ and G_αq_ signal transductions by ligand (Peterson & Luttrell, 2017). Arrestin is also known to work as a mediator as well as GPCR desensitization. For example, FFAR4/GPR120 and Arrestin 3 influence to anti‐inflammation via transforming growth factor‐ß activated kinase 1 (TAK1) and TAK1 binding protein (TAB1) (Oh et al., 2010). In FFARs, long‐chain fatty acids dose‐dependently promote the recruitment of Arrestin‐2 and ‐3 to human FFAR1/GPR40 (Mancini et al., 2015; Qian et al., 2014). The recruitment of Arrestins has been confirmed under physiological conditions, chronic exercise–activated Arrestin‐3 with FFAR4/GPR120, and decreased inflammatory responses in mice (Gaspar et al., 2019). In the present study, we examined the interaction between four types of cat arrestins with FFAR2 and FFAR3 using the NanoBiT assay, and our results revealed that both cat FFAR2 and FFAR3 interacted with Arrestin‐3/β‐arrestin‐2. Interestingly, not only did human FFAR2 and FFAR3 interact with Arrestin‐3, but these receptors also formed a heterodimer in colon epithelial cells (Ang, Xiong, Wu, & Ding, 2018). In addition to G_αi_ and G_αq_ interaction, the heterodimer of FFAR2 or FFAR3 and Arrestin‐3 mediated the ligand signal to p38 phosphorylation. It is unknown whether cat FFAR2 or FFAR3 and Arrestin‐3 intervene in the signal transduction from SCFA in colon epithelial cells, but they may regulate some physiological events such as intestinal inflammation and metabolic regulation.

## 5 CONCLUSIONS

In conclusion, we identified FFAR2 and FFAR3 in cats, which are characterized as G_αi/o_ receptors and FFAR2 also act as G_αq_ receptor. We observed the highest mRNA expression of FFAR2 in the bone marrow and the highest mRNA expression of FFAR3 in the spleen. The NanoBiT assay revealed that desensitization of cat FFAR2 and FFAR3 was induced by binding of Arrestin‐3. FFAR2 and FFAR3 may play some roles in carnivorous cats, and it is suggested that both FFAR2 and FFAR3 are involved in lipid metabolism as SCFAs biosensors.

## CONFLICT OF INTEREST

The authors declare no conflict of interests related to this work.

## AUTHOR CONTRIBUTION

**Koh Kawasumi:** Formal analysis. **Kozo Ohkusu‐Tsukada:** Formal analysis; Funding acquisition. **Toshiro Arai:** Project administration.

### PEER REVIEW

The peer review history for this article is available at https://publons.com/publon/10.1002/vms3.356.
